# Complex regression Doppler optical coherence tomography

**DOI:** 10.1117/1.JBO.23.4.046009

**Published:** 2018-04-27

**Authors:** Sahar Elahi, Shi Gu, Lars Thrane, Andrew M. Rollins, Michael W. Jenkins

**Affiliations:** a Case Western Reserve University, Department of Pediatrics, Cleveland, Ohio, United States; b Case Western Reserve University, Department of Biomedical Engineering, Cleveland, Ohio, United States

**Keywords:** optical coherence tomography, Doppler optical coherence tomography, high-speed optical coherence tomography, MHz Fourier domain mode locked laser, hemodynamics

## Abstract

We introduce a new method to measure Doppler shifts more accurately and extend the dynamic range of Doppler optical coherence tomography (OCT). The two-point estimate of the conventional Doppler method is replaced with a regression that is applied to high-density B-scans in polar coordinates. We built a high-speed OCT system using a 1.68-MHz Fourier domain mode locked laser to acquire high-density B-scans (16,000 A-lines) at high enough frame rates (∼100  fps) to accurately capture the dynamics of the beating embryonic heart. Flow phantom experiments confirm that the complex regression lowers the minimum detectable velocity from 12.25  mm/s to 374  μm/s, whereas the maximum velocity of 400  mm/s is measured without phase wrapping. Complex regression Doppler OCT also demonstrates higher accuracy and precision compared with the conventional method, particularly when signal-to-noise ratio is low. The extended dynamic range allows monitoring of blood flow over several stages of development in embryos without adjusting the imaging parameters. In addition, applying complex averaging recovers hidden features in structural images.

## 1 Introduction

Doppler optical coherence tomography (OCT) is a functional extension of OCT, which estimates velocity by detecting the Doppler frequency change imposed on OCT light by moving scatterers.^1^^,^^2^ The range of velocities measured by Doppler OCT is dictated by the imaging speed of the system and the applied scanning pattern, which makes it difficult to accommodate certain samples. For example, when imaging vascular networks, only certain velocities fall within the detectable range as blood velocity can vary from ∼100  μm/s in capillaries^3^ to 200  mm/s in arterioles.^4^ Our group is specifically interested in studying the hemodynamics of early stage embryonic hearts. Several groups have implemented and developed OCT Doppler imaging for studying embryonic heart development.^5^^–^^20^ Hemodynamics and wall motion undergo significant increases in velocity as the embryonic heart develops. Experimental studies indicate that altered hemodynamics in early stage embryonic hearts can lead to congenital heart diseases, motivating close monitoring of blood flow over several stages of development.^21^^–^^30^ As a result, imaging and processing parameters are adjusted continuously to accommodate the large velocity range required in longitudinal cohort studies of embryos.

Several groups have achieved a more desirable velocity range by modifying the time interval over which the Doppler phase shift is measured. The minimum resolvable phase is dictated by the phase stability of the system and the maximum phase is confined to ±π to avoid ambiguity caused by phase wrapping. Conventionally, the phase difference is measured between two adjacent A-lines, where the time interval is the inverse of the imaging speed. Increasing the time interval improves the minimum detectable velocity at the expense of A-line rate, which may not be desirable for *in vivo* imaging applications. Alternatively, the phase shift measurement can be applied to nonadjacent A-lines while maintaining the spatial correlation to increase the time interval. For example, B-scan Doppler measures the phase shift between the same A-line from consecutive B-scans, which increases the time interval to the acquisition time of one B-scan.^31^^–^^33^ Although B-scan Doppler enables slow velocity detection, phase wrapping occurs more quickly, which limits detection of the higher velocities. Other groups demonstrated a tunable velocity range by applying varying scanning protocols^34^ or employing a dual-beam setup that uses two spatially offset beams,^35^^,^^36^ which requires prior knowledge of the velocity range within the sample to set the parameters and adds complexity to the system. The above-mentioned methods merely offer tuning of the velocity range without extending it. An alternative method of velocity estimation is joint spectral and time-domain OCT (STdOCT), where two Fourier transformations are applied in opposite directions.^37^^,^^38^ This approach performs better in low SNR conditions, where phase instabilities are more pronounced. However, the velocity resolution of the measurement is defined by the number of temporal samples, which can require a large number of A-lines when performing *in vivo* studies.

Recent advances in swept laser sources for OCT imaging have enabled multi MHz A-line rates that open up possibilities for Doppler imaging.^39^ The fastest commercial swept laser source is a 1.6-MHz Fourier domain mode locked laser (FDML) from Optores GmbH, Germany, which is based on lasers developed in the Huber Lab.^40^^–^^42^ Using this laser, Wang et al.,^43^ demonstrated direct four-dimensional (4-D) imaging of cardiovascular structure in live mouse embryos at a volume rate of ∼43  Hz. Measurements of the wall motion were presented using the 4-D data and B-scan Doppler was applied to quantify the blood flow velocity. Zhi et al.^44^ performed three-dimensional (3-D) and 4-D imaging of microcirculation within tissue beds *in vivo*. Optical microangiography (OMAG) was achieved using B-scan Doppler without any motion correction owing to the ultrahigh imaging speed of the FDML laser. Wei et al.^45^ demonstrated a volumetric OMAG method (vOMAG) using intervolume analysis to monitor blood flow in the mouse brain *in vivo*. Direct 4-D imaging at a rate of 200  volumes/s allowed measurements of slow blood flows in capillary vessels. These papers all use the extra speed to decrease the Doppler time interval between B-scans or volumes, but still have a relatively low-velocity ceiling before phase wrapping occurs.

Instead of tuning the velocity range, the ultrafast A-line rate offered by this laser can be traded off to enable a multipoint Doppler calculation by obtaining densely sampled B-scans. Having access to multiple data points from the same location allows for new phase measurement methods that could possibly overcome some limitations of the conventional method. In this paper, we introduce a new complex regression method of measuring Doppler phase shifts. We built a high-speed OCT system using the 1.6-MHz FDML laser to acquire high-density B-scans (16,000 A-scans) while still achieving high frame rates (100 fps). In comparison to conventional Doppler processing (finding a two-point difference), our flow phantom experiments demonstrate that complex regression extends the dynamic range, and provides higher accuracy and precision. The complex regression method is also demonstrated in live quail embryo hearts.

## 2 Methods

### 2.1 Complex Regression Doppler Optical Coherence Tomography

**Fig. 1:**
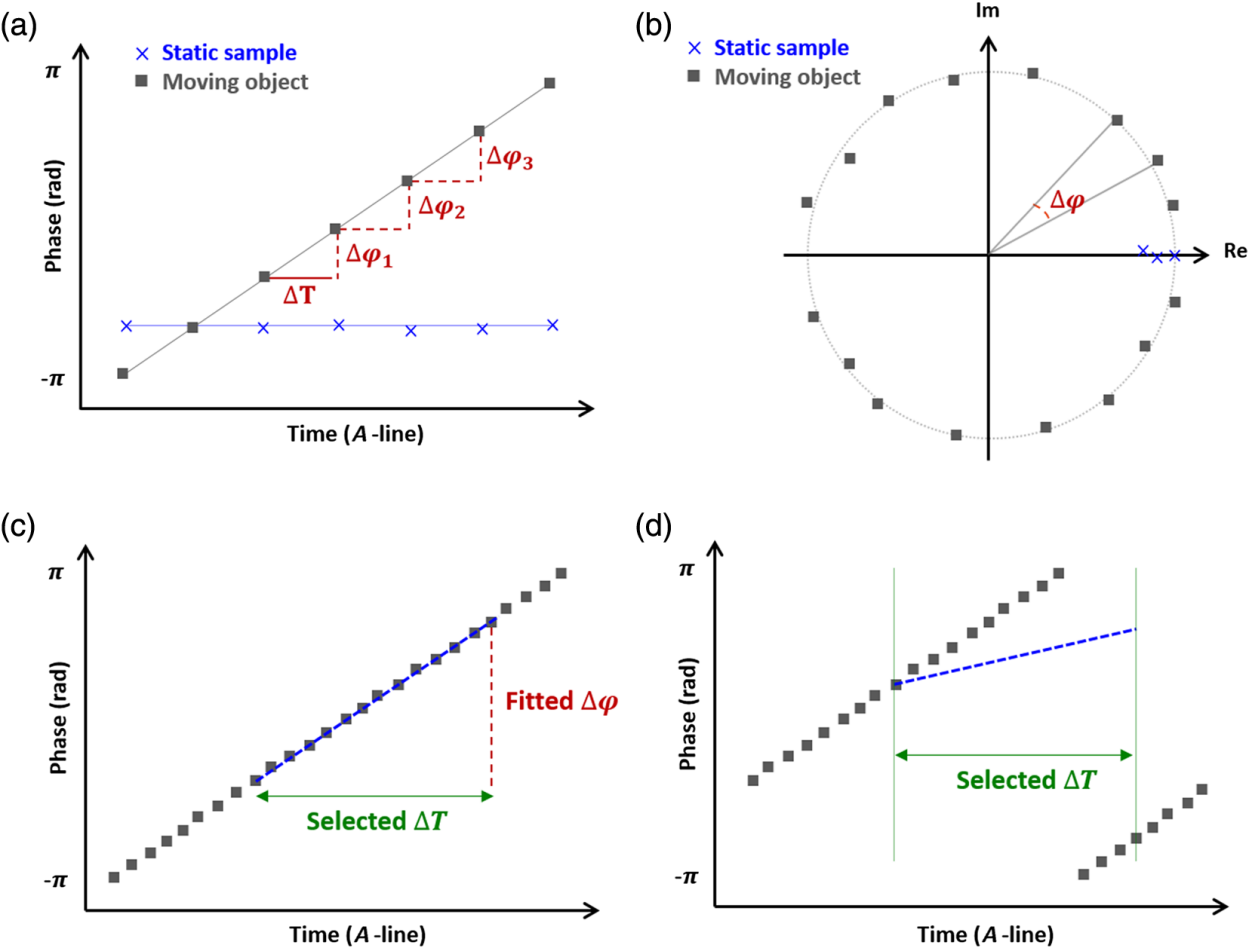
(a) Progression of phase over time at a set location in rectangular coordinates: blue is a static sample, black is a moving object at a constant velocity, where the change in phase is accurately measured at multiple locations (red). (b) Progression of phase over time at a set location in polar coordinates: blue is a static sample, black is a moving object at a constant velocity, (c) Measuring phase shift by fitting a line (blue), and (d) occurrence of phase wrapping within the time interval causes fitting to fail.

The conventional Doppler OCT method calculates velocity by determining the phase difference between two adjacent A-lines. Initially, the phase difference was computed by subtracting the phase of subsequent A-lines, but it has become more common to perform the calculation directly on the complex A-lines to enhance the SNR. Figure 1(a) shows the progression of phase over time for a set location in rectangular coordinates. If the sample is static [Fig. 1(a): blue], the phase remains constant. When there is movement, the phase accumulates over time at a rate corresponding to the speed of the moving object [Fig. 1(a): black]. Figure 1(b) shows the complex data points in polar coordinates, where the phase either remains constant for static objects [Fig. 1(b): blue] or circles around the origin as phase accumulates for objects in motion [Fig. 1(b): black].

Typically, multiple two-point differences are averaged together to compute the velocity, but utilizing several points to fit the phase shift could improve the accuracy and extend the dynamic range of Doppler OCT. In theory, fitting the data offers superior performance in comparison to averaging, although is computationally more expensive. The simplest form of fitting is to implement a linear regression on the data points as shown in Fig. 1(c). Taking measurements over longer intervals leads to increased phase accumulation and possibly phase wrapping, in which case, fitting fails in rectangular coordinates [Fig. 1(d)]. Although applying corrections should be feasible, phase wrapping can be mainly avoided in polar coordinates as phase accumulates in a circle over long intervals. Hence, phase wrapping is independent of the phase measurement interval and only occurs if phase accumulation exceeds 2π within the time interval of two successive A-scans. To the best of our knowledge, a simple fitting scheme in polar coordinates does not exist.

**Fig. 2:**
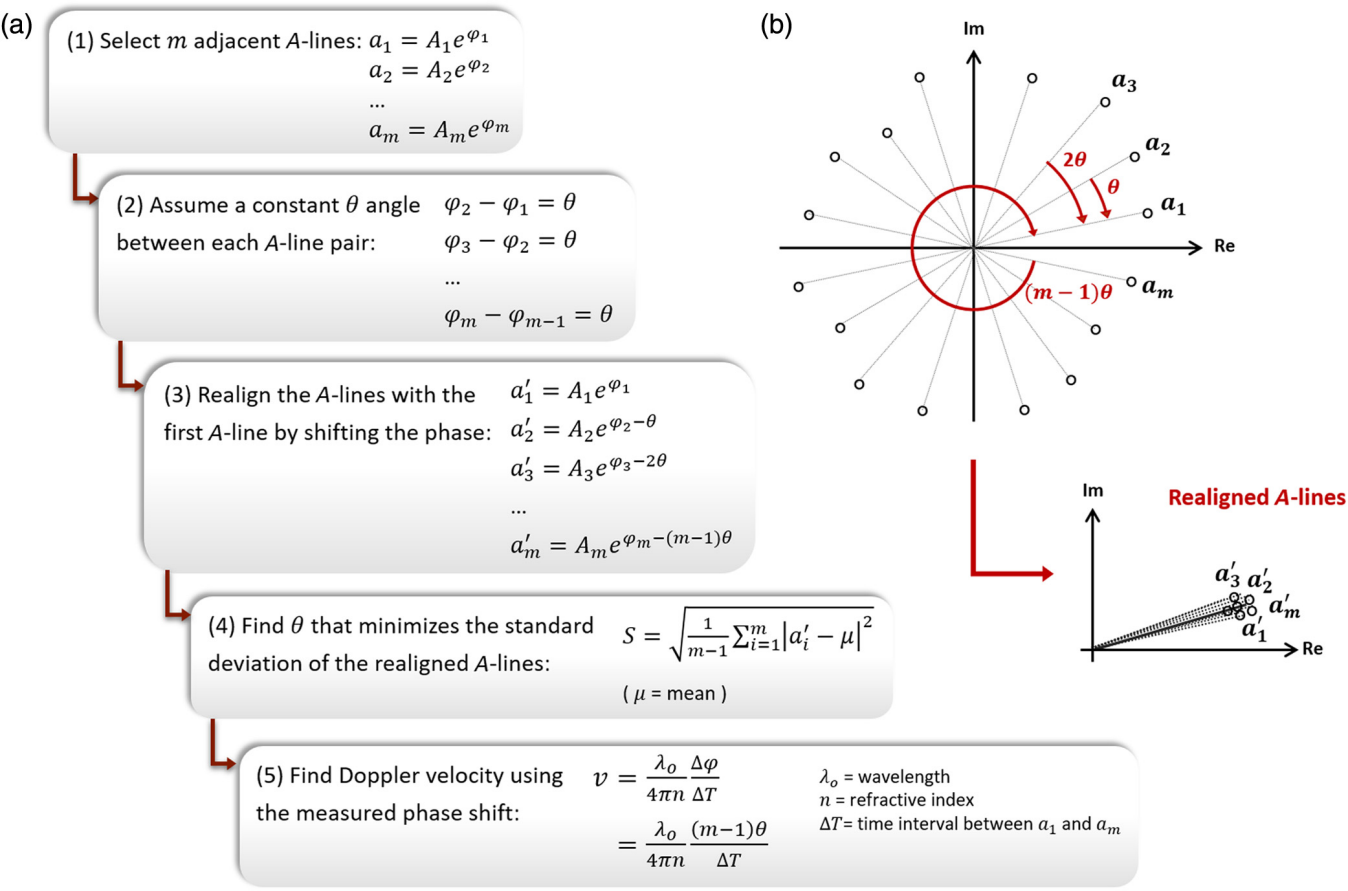
Complex regression Doppler: (a) the steps of complex regression method to measure the phase shift, (b) complex signal of m adjacent A-lines with a constant phase difference of θ (top), realigned A-lines with the first A-line (bottom).

We present a fitting method for measuring Doppler phase shifts that is only achievable in polar coordinates. The steps of complex regression Doppler are given in Fig. 2 along with an illustration of the data in polar coordinates. Step 1 in Fig. 2 shows the complex signal (amplitude and phase) of m adjacent A-lines. With this method, ΔT represents the time interval between the first and last A-scans instead of the time interval between adjacent A-scans. Step 2 in Fig. 2 assumes that the angle θ between each pair is nearly constant. Typically, the velocity is considered constant during the measurement window in Doppler OCT imaging, although this assumption is not always valid. To determine the phase shift, sequentially increasing multiples of θ are subtracted from the phase of each point in attempt to align all points with the first A-scan (Fig. 2, step 3). Under ideal conditions, A-scans would be aligned perfectly; however, the presence of noise causes slight variations in the phase. Furthermore, as particles move through the imaging window, backscattering changes and leads to fluctuations in the signal amplitude. The objective of this method is to find the θ value from [−π to π] that minimizes the standard deviation of the shifted points (Fig. 2, step 4). The corresponding phase shift that is defined as Δφ=(m−1)θ is used to obtain the Doppler velocity (Fig. 2, step 5). Complex regression acts like a weighted regression by taking into account the amplitude of the data points as well as the phase. Higher amplitude signals are associated with more accuracy as phase sensitivity is inversely proportional to the square root of the signal-to-noise ratio (SNR). Moreover, errors in lower amplitude signals create smaller phase measurement errors when doing regression on the complex data.

### 2.2 Imaging Setup

**Fig. 3:**
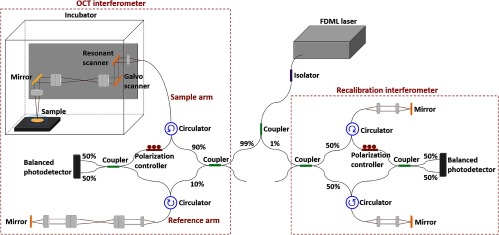
Schematic of the high-speed OCT system.

Our high-speed OCT system (Fig. 3) consists of an FDML swept laser source (Optores GmbH, Germany) operating at a sweep rate of 1.68 MHz, with a center wavelength of 1315 nm, a tuning range of 110 nm, and a 6-dB falloff at a depth of 2.5 mm in air. The OCT interferometer was built in a Mach–Zehnder configuration, where the sample arm was placed inside an incubator to control the temperature and humidity during imaging of live quail embryos. The reference arm includes the same set of lenses used in the sample arm to correct for dispersion. A second interferometer was used to obtain a recalibration signal for k-space resampling of the OCT fringes. The interference signals were acquired by two 1.6-GHz dual balanced photodetectors (Thorlabs Inc.) and a 12 bit, 4  GS/s digitizer (Alazar Technologies Inc., Canada). The beam is scanned by a resonant scanner (Electro-Optical Products Corp.) at a fixed frequency of 3.59 kHz along the fast axis and a galvanometer scanner (Cambridge Technology) along the slow axis. The axial resolution is ∼12  μm in air and the beam spot size at the imaging focal plane is ∼15  μm over a $4\times 4\;{\mathrm{mm}}^{2}$ field of view. The measured sensitivity was ∼103  dB and the phase stability was 0.096 rad for M-mode imaging. The detectable phase range of [0.096,π] and time interval between adjacent A-lines of 594 ns result in the velocity ranges of 12.25 to 401.05  mm/s for the conventional Doppler method, respectively.

### 2.3 Validation Experiments

To evaluate the new complex regression method for Doppler, 2% lipid solution (Intralipid, Clayton, North California) was pumped through a capillary tube with an inner diameter of 300  μm using an NE-300 Just Infusion™ syringe pump (New Era Pump Systems Inc.). The angle between the capillary tube and the imaging beam was set to ∼80  deg and the axial velocity was varied from 84  μm/s to 400  mm/s. The velocity values were evenly spread out in logarithmic scale, corresponding to: 51, 84, 138, 374, 616  μm/s; 1.01, 1.66, 2.74, 4.51, 7.43, 12.23, 20.13, 33.13, 54.53, 89.74, 147.69, 243.05, and 400  mm/s. M-mode images were acquired while the beam was positioned in the middle of the capillary tube. Two sets of measurements were taken: high SNR conditions representing strong superficial signals and low SNR conditions, where sample power was reduced by 20 dB using a neutral density filter to represent weak signals reflected from deep within the tissue.

Complex regression Doppler was also used for *in vivo* measurement of blood flow in the hearts of quail embryos. Fertilized quail eggs were incubated in a humidified incubator (Eppendorf New Brunswick, Germany) at 38°C. At 48 h of development, the eggshells were removed and the embryos were cultured in Petri dishes. Tubular hearts of the embryos were imaged at 48 and 72 h of development.

Complex regression Doppler was implemented in MATLAB R2016a (MathWorks Inc.), running on a 2.20 GHz, Windows 10 workstation. Each frame consisted of 16,000×594  pixels (acquisition time 9.52 ms), where M-scans corresponded to a fixed position and B-scans were acquired over a lateral length of 1 mm. B-scans were acquired by scanning with the galvanometer mirror while keeping the position of the resonant scanner fixed. As our previous Doppler experiments suggested a sampling rate of 3× the lateral spot size or greater is needed,^14^^–^^16^^,^^46^ we selected 64 points for each regression which corresponded to 4-μm lateral sampling (ΔT=38  μs). Within each group of 64 A-lines, θ is changed in increments of 1 mrad and the standard deviation is computed. The θ that minimizes the standard deviation is reported to determine the phase shift (Δφ=63θ). The performance of complex regression was compared to the conventional Doppler method, where a large time interval allowed for more averaging. For any given time interval of 64 A-lines, the phase was measured by complex regression $\Delta \varphi_{\mathrm{CmpReg}}$, the conventional Doppler method where the first five measurements are averaged $\Delta \varphi_{\mathrm{CnvN}5}$, and the conventional Doppler method where all 63 measurements are averaged $\Delta \varphi_{\mathrm{CnvN}63}$. Measured phase shifts were used to find the axial velocity, where $\lambda_{o}=1315\;\mathrm{nm}$, n=1.38, and ΔT=594  ns. Absolute velocity was estimated by correcting for the Doppler angle.

Doppler images of the quail embryos were rendered in Amira 6.0.1 (FEI, Thermo Fisher Scientific Inc.) to visualize the beating heart and the blood flow over time.

## 3 Results

**Fig. 4:**
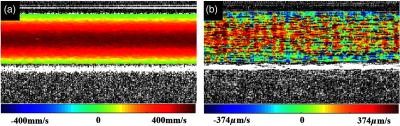
Extended velocity range using complex regression Doppler to measure the phase shift: M-mode Doppler images of flow phantom pumped at an axial velocity of (a) 400  mm/s (center of tube) and (b) 374  μm/s (center of tube).

Flow phantom experiments were performed to demonstrate the extended Doppler range and evaluate the performance of complex regression in comparison to the conventional method. OCT images of the flow phantom at the speeds of 400  mm/s and 374  μm/s are shown in Fig. 4, where complex regression Doppler was applied to estimate the velocity. Complex regression of 64 A-lines extends the lower end of the velocity range to 374  μm/s, whereas the higher end is detected without phase wrapping.

**Fig. 5:**
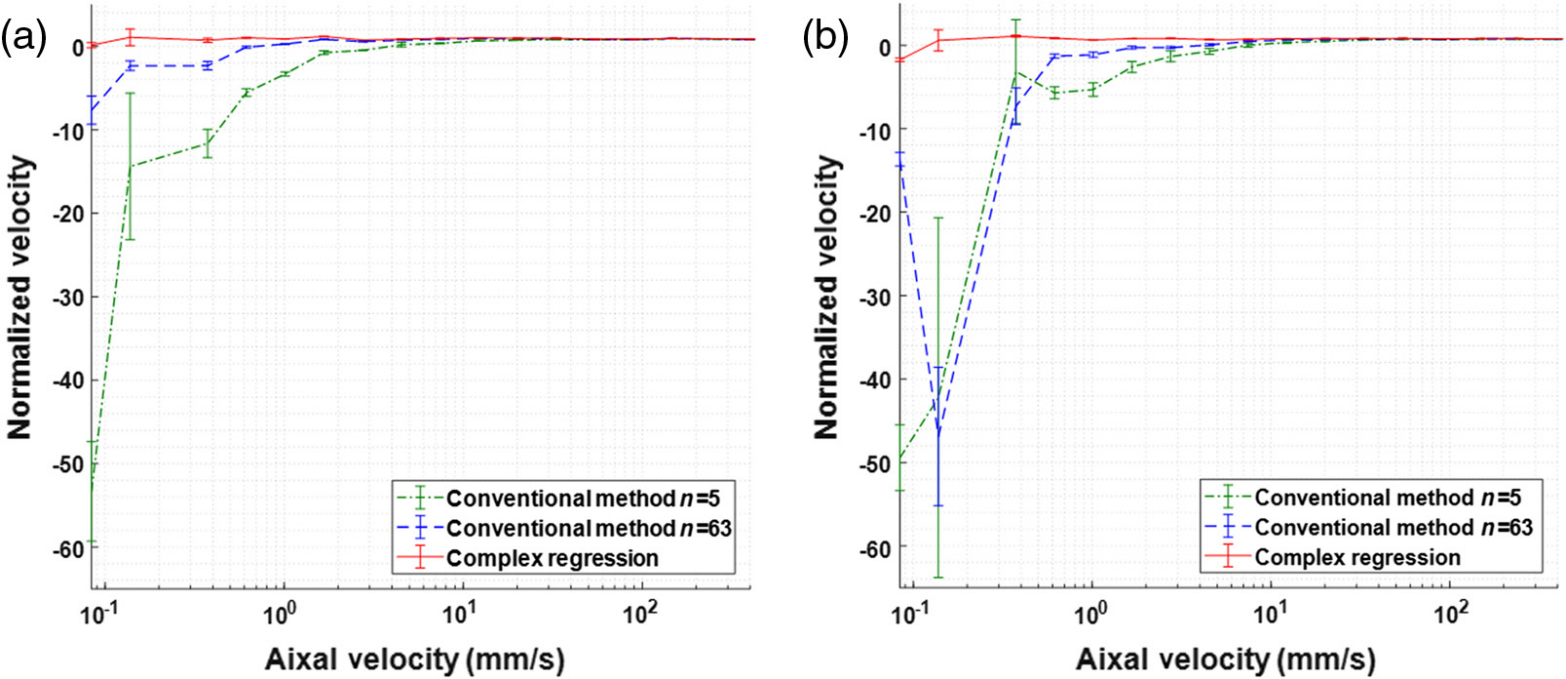
Normalized velocity vs axial velocity (log scale): (a) high SNR M-scan, (b) low SNR M-scan, green: conventional method using five averages $\Delta \varphi_{\mathrm{CnvN}5}$, blue: conventional method using 63 averages $\Delta \varphi_{\mathrm{CnvN}63}$, red: complex regression $\Delta \varphi_{\mathrm{CmpReg}}$, and bars: standard error.

Figure 5 shows the comparison between complex regression Doppler and the conventional method when measuring velocities within the range of 85  μm/s to 400  mm/s. The phase shift in the M-mode images was measured by complex regression $\Delta \varphi_{\mathrm{CmpReg}}$, the conventional method with five averages $\Delta \varphi_{\mathrm{CnvN}5}$, and the conventional method with 63 averages $\Delta \varphi_{\mathrm{CnvN}63}$. The measured phase shifts were converted to velocities and the mean at the peak of the flow profile was calculated across 250 lines of the image. The measured velocities were normalized by the actual velocities to better visualize the accuracy over the wide range. The standard error (the ratio of the standard deviation by the square root of the number of samples) is included to show the precision of the measurements. As averaging reduces the noise by a factor of $1/\sqrt{n}$,^47^ using 5 and 63 averages with the conventional method is expected to lower the minimum detectable velocity to ∼5.5 and ∼1.5  mm/s, respectively. Moreover, velocity has an inverse relationship with the time interval ΔT and using 64 A-lines in the complex regression is expected to lower the minimum detectable velocity to ∼194  μm/s. When SNR is high [Fig. 5(a)], the minimum detectable velocities using $\Delta \varphi_{\mathrm{CnvN}5}$, $\Delta \varphi_{\mathrm{CnvN}63}$, and $\Delta \varphi_{\mathrm{CmpReg}}$ are 7.43  mm/s, 1.66  mm/s, and 374  μm/s, respectively. In the low SNR condition [Fig. 5(b)], a neutral density filter was used to reduce the sample power by 20 dB, lowering the SNR by a factor of 10. As the phase noise is inversely affected by the SNR ($1/\sqrt{\mathrm{SNR}}$),^48^ the minimum detectable velocities are expected to increase about 3.16 times. The minimum velocities detected by $\Delta \varphi_{\mathrm{CnvN}5}$, $\Delta \varphi_{\mathrm{CnvN}63}$, and $\Delta \varphi_{\mathrm{CmpReg}}$ are 20.13  mm/s, 7.43  mm/s, and 616  μm/s, respectively.

**Fig. 6:**
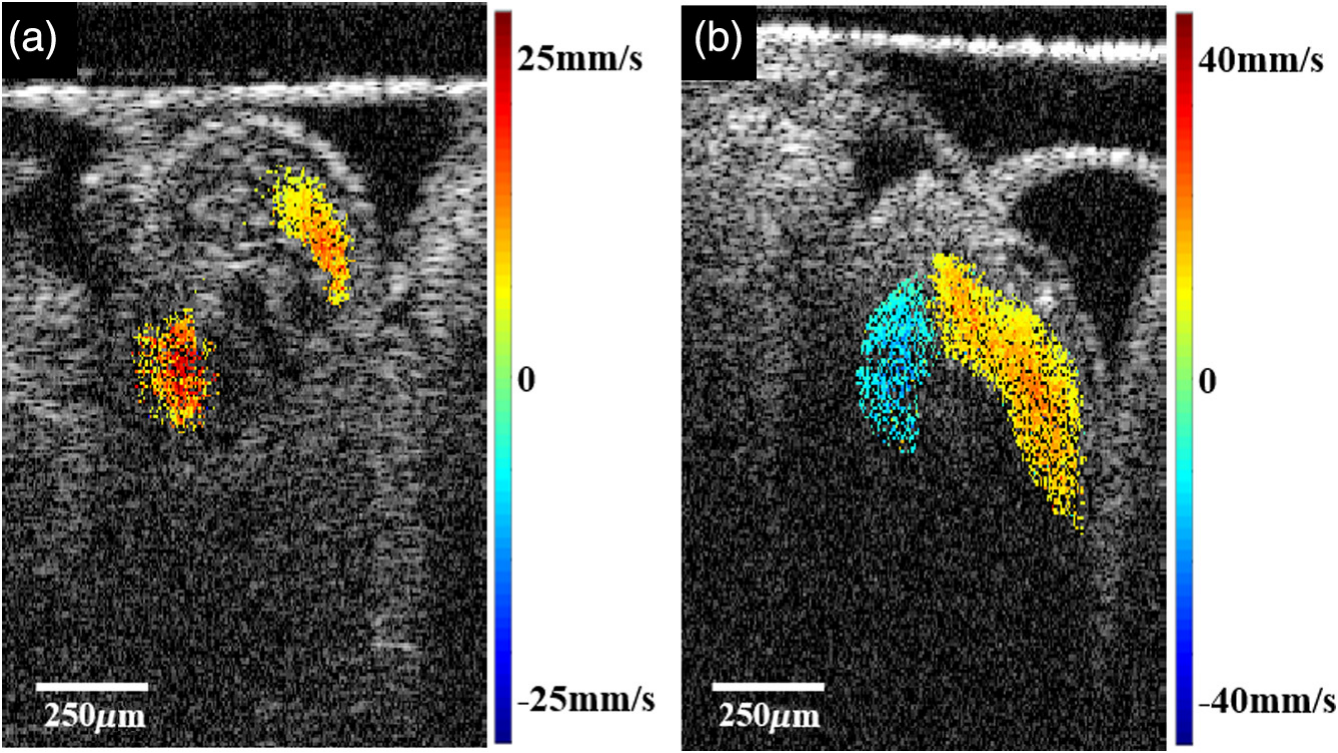
Example of a longitudinal study without changing imaging parameters: (a) structural image of day 2 quail embryo overlaid by Doppler image (Video 1), (b) structural image of day 3 quail embryo overlaid by Doppler image. The complex regression allows detection of varying velocity ranges at different stages of development (Video 2). (Video 1, QuickTime, 4.2 MB [URL: https://doi.org/10.1117/1.JBO.23.4.046009.1]; Video 2, QuickTime, 4.6 MB [URL: https://doi.org/10.1117/1.JBO.23.4.046009.2]).

An example of using complex regression in longitudinal embryo studies is shown in Fig. 6, where the imaging parameters remain unchanged at different stages of embryonic development (movies are shown in Video 1 and Video 2). Images of the tubular heart of a quail embryo were acquired on day 2 and day 3, where the maximum values of the measured Doppler velocity was 24  mm/s [Fig. 6(a)] and 39  mm/s [Fig. 6(b)], respectively.

**Fig. 7:**
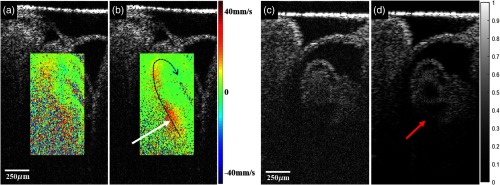
Structural images of the tubular heart of a quail embryo (coronal view) overlaid by Doppler images: (a) using five A-lines (conventional method) (b) using 64 A-lines (complex regression). Black dotted curves illustrate the direction of blood flow, white arrow points to deeper part of the flow that is missing in (a). Structural images of tubular heart of quail embryo (transverse view): (c) no averaging, and (d) complex averaging. Red arrow points to the back wall of the heart that is not seen clearly in (c).

Figure 7 shows Doppler imaging of blood flow and structures with high-density B-scans. Figures 7(a) and 7(b) show the tubular heart of a quail embryo, where the direction of blood—flowing to the left and upward into the tube and to the right and downward out—is depicted by the black dotted curve. The deeper part of the flow that is missing by the conventional method is detected [white arrow in Fig. 7(b)] with the complex regression method. Structural images can also benefit from the extra A-lines by performing complex averaging. Figures 7(c) and 7(d) show cross-sectional images of the tubular heart, where the back wall is discernible only in the complex averaged image [red arrow in Fig. 7(d)].

## 4 Discussion

The limited dynamic range of Doppler OCT can lead to phase wrapping at high velocities or loss of sensitivity at slow velocities, depending on the imaging speed and measurement technique. The variations of the conventional method merely move the limited range to accommodate expected velocities within the sample. We introduce a new method of measuring Doppler phase shift to extend the dynamic range using high-density B-scans. The method applies a regression in polar coordinates, where the phase circles around the coordinates and phase jumps are not observed at the phase wrapping points. We used 64 A-lines in our complex regression to measure the phase shift, which improved the minimum detectable velocity from 12.25  mm/s to 374  μm/s, whereas the maximum velocity of 400  mm/s was detected without phase wrapping.

The performance of complex regression was compared to the conventional Doppler method for high and low SNR signals. When SNR is high, complex regression and the conventional method using 63 averages show similar improvement in precision and accuracy; however, complex regression extends the range further. When SNR is low, complex regression exhibits better precision and accuracy at lower velocities compared to the conventional method using 5 and 63 averages, which indicates it is a superior method for weaker signals that are reflected from deep within the tissue. We also applied the conventional Doppler method to estimate the velocity by finding the phase shift between the first and last A-lines as ΔT was increased (data are not presented). As expected, a longer ΔT improved the minimum detectable velocity, but not as much as complex regression or averaging. Additionally, the upper limit caused by phase wrapping decreased with a longer ΔT. Complex regression produces accurate and precise results while extending the Doppler range.

Our experimental results are confirmed by the theory when comparing complex regression to the conventional Doppler method using more averages. Averaging reduces the noise by a factor of $1/\sqrt{n}$,^47^ which helps with detection of lower velocities. Increasing the number of averages in the conventional method to 63 is expected to improve the minimum detectable velocity by approximately eightfold. Moreover, velocity has an inverse relationship with the time interval ΔT and using 64 A-lines in the complex regression is expected to lower the minimum detectable velocity by ∼63 fold. The SNR also plays a role in dictating the minimum detectable velocity as phase noise is effected by $1/\sqrt{\mathrm{SNR}}$.^48^ Therefore, reducing the SNR by 20 dB in our experiments is expected to increase the minimum detectable velocity by ∼3.16 fold. Although our data are in agreement with the theoretical values, the measured velocities did not always match the precise numbers as the very large velocity range of these experiments was undersampled. For example, the minimum velocity detected by the complex regression method was only fourfold better than the conventional method using 63 averages instead of 7.8-fold because the theoretical minimum (194  μm/s) falls in between our sampled velocities (138 and 374  μm/s). In summary, while averaging greatly reduces the noise, fitting the data has a lower minimum detectable velocity and measurements taken in the low SNR condition demonstrate a similar trend to that of the high SNR condition.

The variation in the phase noise of the laser during different measurements could be a contributing factor when extending the velocity range. The minimum detectable velocity can be further improved by correcting the phase noise. Chen et al.^49^ reported lowering the minimum detectable velocity from 1.01  mm/s to 268.2  μm/s by reducing the phase noise using an FBG filter and spectral phase encoding. We plan to add a glass slip above the sample as a reference for numerical phase correction to remove phase variations over time as well as the differences among the copies of the fundamental sweep of the buffered FDML laser.^42^

The extended dynamic range is valuable when conducting longitudinal studies on embryos, where the stage of development and the orientation of the heart tube affect the range of Doppler phase shifts. The low velocities are desired to detect the slow blood flows near the wall for shear stress estimation as well as measuring the movement of the wall. High velocities are needed to determine the maximum blood flow velocity. Although complex regression does not cover our entire range, the offered range is larger than what is available with the conventional method or the tuning strategies. Moreover, complex regression eliminates the need for prior knowledge of velocity range and allows monitoring of blood flow over several stages in a cohort of embryos without adjusting the imaging parameters.

High-density B-scans enable application of complex averaging to enhance structural images. Averaging is very effective when structural information in consecutive A-scans is almost identical and the resolution is not degraded, therefore increasing dynamic range and improving contrast.^50^^,^^51^ When the presence of blood causes additional scattering, complex averaging might retrieve hidden features.^52^ In the example shown in Fig. 7, the back wall of the tubular heart was recovered in the complex averaged image, making it possible to visualize the entire structure of the embryonic heart from OCT images. Likewise, Doppler images generated by complex regression contain deeper flows that go undetected using the conventional method.

We chose a direct approach by densely sampling Δφ (at 1 mrad) as complex regression is under fixed boundary conditions [−π, π]. This brute force method yielded satisfying results but can easily be improved in the future. The processing time of one B-scan is 150 min due to the required large amount of memory. Implementing more advanced search algorithms such as gradient descent can increase the speed by orders of magnitude. Nonetheless, graphics processing units (GPU) can significantly reduce the processing time of computationally expensive methods. GPU implementation of real-time 3-D structural and Doppler processing have been demonstrated for ultrahigh-speed OCT systems.^53^^,^^54^ Taking advantage of commercially available GPUs with advanced search algorithms, it may be feasible to achieve real-time complex-regression Doppler OCT in the future.

In conclusion, we have demonstrated a new method of measuring Doppler phase shifts by capturing high-density B-scans. The complex regression Doppler extends the dynamic range by lowering the minimum detectable velocity while providing higher accuracy and precision compared to the conventional method. Also, high-density B-scans enable the application of complex averaging to recover hidden features in structural images. The complex regression eliminates the need for prior knowledge of velocity range within the sample, which could be over three orders of magnitude and allows monitoring of blood flow in longitudinal embryonic studies without adjusting the imaging parameters.
